# Advances in Skin Wound and Scar Repair by Polymer Scaffolds

**DOI:** 10.3390/molecules26206110

**Published:** 2021-10-10

**Authors:** Shuiqing Zhou, Qiusheng Wang, Ao Huang, Hongdou Fan, Shuqin Yan, Qiang Zhang

**Affiliations:** State Key Laboratory of New Textile Materials and Advanced Processing Technologies, School of Textile Science and Engineering, Wuhan Textile University, Wuhan 430200, China; zsqdaisy@163.com (S.Z.); qiusheng-wang@hotmail.com (Q.W.); haaaaa0408@163.com (A.H.); hddddd1206@163.com (H.F.)

**Keywords:** wound healing, scar inhibition, polymer scaffolds, oxygen generating scaffolds, stem cells, growth factors

## Abstract

Scars, as the result of abnormal wound-healing response after skin injury, may lead to loss of aesthetics and physical dysfunction. Current clinical strategies, such as surgical excision, laser treatment, and drug application, provide late remedies for scarring, yet it is difficult to eliminate scars. In this review, the functions, roles of multiple polymer scaffolds in wound healing and scar inhibition are explored. Polysaccharide and protein scaffolds, an analog of extracellular matrix, act as templates for cell adhesion and migration, differentiation to facilitate wound reconstruction and limit scarring. Stem cell-seeded scaffolds and growth factors-loaded scaffolds offer significant bioactive substances to improve the wound healing process. Special emphasis is placed on scaffolds that continuously release oxygen, which greatly accelerates the vascularization process and ensures graft survival, providing convincing theoretical support and great promise for scarless healing.

## 1. Introduction

Hyperplastic scars and keloids are pathological scarring caused by defects that arise during the normal wound healing process [1]. Hypertrophic scars occur after a deep burn injury or trauma, with a prevalence of approximately 70% [2]. In contrast to normal scar, the hyperplastic scar is characterized by protruding skin surface, irregular shape, flushing and hyperemia, hardness, and difficulty in reproducing some components of nature skin such as subepidermal appendages, hair follicles, or glands. In addition to aesthetic defects, pathological scars may cause pain, itch, deformities, contractures, and impairment of tissue other functions. Different from hypertrophic scars, keloids grow continuously beyond the boundaries of the original wound. Genetic, genetic, and immune are the major factors in the formation of keloids [3]. Thus, hyperplastic scars served as the main research object in this review.

To coordinately regulate the wound repair, cytokines, growth factors, and extracellular matrix (ECM) in host tissue perform different phenotypes, morphologies, and functions. However, the etiology and pathogenesis of hyperplastic scars have no consistent conclusions yet [4]. Superficial injuries never contribute to the formation of scars. When severe damage occurs in the dermis, upregulation of pro-inflammatory factors and conversion of fibroblasts to myofibroblasts occur [5]. Excessive deposition and disorderly arrangement of collagen fibers [6] and overproduction and deposition of ECM proteins [7] imply upcoming scar formation. Unlike adult wounds, fetal wounds can be completely regenerated and scarless healing in the first and second trimesters of pregnancy, accompanied by fewer collagen deposition, higher content of hyaluronic acid (HA), and weaker inflammation response [8]. In adulthood, wound healing can only be achieved through the deposition and remodeling of collagen, and most of the wounds can be healed at the cost of scar formation.

The conventional treatments for scar repair mainly focus on passive interventions such as surgical therapy, pressure therapy, radiation therapy, the introduction of drugs, and laser therapy. However, surgical therapy may put patients with severe trauma in an embarrassing situation of “not enough donors”, and new scars may also be formed due to the slow recovery and weak traumatic sutures in the autologous donor area [9]. Pressure therapy may directly or indirectly reduce collagen deposition [10]. However, the norms of pressure therapy come from clinical experience, with a lack of reasonable and strict controlled studies. Individual differences, the heterogeneous pressure distribution, joint movement, and skin surface irregularities will affect the treatment effect and even cause complications [11]. The existence of radioactive sources (doses ≥ 30 Gy) has sparked debate about the safety of radiation therapy, such as triggers fatal complications such as visceral injuries [12]. For pharmacological interventions, the combination of glucocorticoid triamcinolone acetonide and 5-fluorouracil is instrumental in mitigating the pain and itching of scar, accompanied by a lower recurrence rate and fewer side effects [13]. Laser treatment improves the thickness, pain, flexibility, pigmentation, and itching of the scar, with a fast recovery period. Yet, the effect of laser treatment is also limited to a certain extent by individual difference [14]. To sum up, conventional clinical strategies, which are passive repair after scar formation, which is difficult to remove scarring at the root, accompanied by limitations and incompleteness and the risk of triggering complications.

Since the concept of scarless healing was introduced in 1971 [15], scientists have carried out in-depth research on the possibilities of tissue engineering interventions to modulate the wound microenvironment and scar inhibition. By using natural, synthetic, or semi-synthetic tissue-mimetic substitutes to repair damaged or diseased tissues, tissue engineering interventions can effectively modulate the signaling stimuli, components, and cytokines in the ECM, as well as collagen deposition and spatial morphology. Specific means of action commonly used are: promoting angiogenesis, diminishing inflammatory response, influencing cellular proliferation, and controlling ECM deposition. It is worth mentioning that the regeneration of tissues of microvasculature, hair follicles, and sweat glands remains an important challenge for skin regeneration [16,17]. Tissue-engineered scaffolds refer to materials that can be implanted into living organisms after combining with living cells and replace the function of the original tissue. As an extracellular matrix analog of skin damaged tissue, scaffolds should exhibit appropriate physical and mechanical properties and be biocompatible, biodegradable, and bioactive [18]. In addition to covering the wounded surface and providing a physical barrier from external stimuli, a properly designed scaffold will act as a template for guiding cell adhesion, proliferation, and differentiation, as well as guiding the formation of intact skin tissue.

In this review, polymer scaffolds are the main research object. Well-designed scaffolds from different sources were used to mimic the ECM, such as proteins or polysaccharides. Further, different substances such as oxygen sources, cytokines, and growth factors were attached to the scaffold material to accelerate wound tissue remodeling. The mechanisms, current status, and potential therapeutic effects are elucidated by analyzing the three basic elements of tissue engineering-scaffold, seed cells, and growth factors-in the performance of wound healing and scar inhibition.

## 2. Natural Scaffold Materials

Proteins and polysaccharides are the main components of ECM. Polysaccharide materials such as chitosan (CS) and HA; protein materials such as collagen, silk fibroin (SF), and gelatin are often prepared as scaffold materials for reconstruction of traumatic tissue and perform in the form of membranes, microspheres, 3D scaffolds, gels, etc. When necessary, scaffold materials were considered to incorporate other biological agents to improve healing and limit scarring [16].

### 2.1. Polysaccharide Scaffold

CS, a natural cationic polysaccharide obtained from chitin, is considered an ideal healing dressing due to its antibacterial, hemostasis, analgesia, biodegradability, and blood compatibility [19]. The CS hydrogel revealed the potential to prevent scar formation through lower levels of α-Smooth muscle actin (α-SMA) expression and a wound closure rate of 93.8 ± 1.4% after 14 days. Further, a smoother and better appearance was highlighted in the SD rat model after co-treatment with carboxymethyl CS and aloe vera gel [20]. Modified N-carboxymethyl CS expressed less inflammation by activating the transforming growth factor-β1 (TGF-β1)/Smad3 signaling in the Wistar rat model [21]. An asymmetrically wettable AgNPs/chitosan, with the hydrophobic upper surface and hydrophilic opposite surface, was proven to achieve complete wound closure at 10 days in the BALB/c mouse model [22]. The spongy nano-Ag/ZnO CS composite dressing revealed excellent wound healing in the BALB/c mouse model, where the advance rate of clotting increased by 64% and the wound closure rate increased by 200% after 6 days than the ZnO ointment gauze group [23]. In a Wistar rat model, CS-sodium alginate polyethylene glycol films modulate early inflammatory responses and stimulate ordered collagen synthesis, without wound adhesion formation [24].

HA, a major component to initiate wound healing [25], exhibits excellent moisturizing capabilities, naturally occurring in ECM tissue in many parts of the body. High concentrations of HA in fetal tissues spontaneously and quickly heal wounds without scars. The concentration of HA in embryo skin is significantly higher than that in adults, in its epidermis and dermis is significantly higher than other parts of the body [26]. In the New Zealand white rabbit model of the full-thickness skin defect, HA hydrogels treatment alleviated the inflammation via promoted the secretion of α-SMA, and optimized vascularization by increased the expression of vascular endothelial growth factor (VEGF) and inhibited scar formation by reducing TGF-β1 level [27]. Filler injection of HA has also been shown in depressed human scar areas to significantly repair the morphology and structure of the scar [28], which may be a valuable option for the treatment of moderate to severe scar [29]. Bleomycin-loaded dissolving microneedles made up of HA, reduced the formation of human dermal hypertrophic due to its excellent aqueous solubility and biocompatibility [30].

### 2.2. Protein Scaffold

Collagen is the major component of clinically conventional artificial skin substitutes such as Integra^®^ and Apligraf [31]. During the wound healing process, abnormal reorganization, irregular arrangement, and excessive deposition of collagen because of fibroblast excessive secretion, contribute to scar formation [32]. Previous studies have demonstrated that the implantation of artificial collagen scaffolds into the damaged tissue to reshape the ECM may be an effective strategy to promote wound healing and inhibit scar formation. By preventing cell membrane contraction at the wound edges and wound shrinkage, the porous collagen scaffold promoted the recovery of damaged skin and peripheral nerves [33]. Collagen is often found in combination with other scaffold materials due to its poor mechanical strength and rapid degradation. Nanocomposite collagen scaffold containing gold nanoparticles improved scaffold stability such as tensile strength, reduced inflammation, and accelerated vascularization in Wistar albino rats [34]. Collagen nanofiber scaffolds containing silver nanoparticles were shown to accelerate wound re-epithelialization and shrinkage in the Wistar rat model [35]. The combined use of probiotics and collagen hydrogel/scaffold in the SD rat model enhanced the ultimate load and stiffness of the wound, and reduced inflammation [36]. ZnO-curcumin nanocomposites embedded in hybrid collagen scaffolds had scar indices similar with uninjured or normal skin in the male albino rat model [37]. Polyurethane membrane/knitted mesh collagen-CS double-layer dermal substitute significantly inhibited wound contracture and facilitated angiogenesis and orderly arrangement of new collagen in the SD rat model [38]. The type I collagen hydrogel scaffold with platelet-rich plasma promoted growth and differentiation of dermal stem cells, leading to sweating gland formation in the SD rat model [39].

Gelatin, a protein obtained by partial hydrolysis of collagen, is also widely used in scaffold material for wound repair. The SF/gelatin electrospun nanofibers loaded with astragaloside IV increased the number of microvessels and regularized the collagen deposition by controlling the drug release rate in the SD rat model [40]. To accurately simulate skin structure (dermal, epidermal, subcutaneous tissue), a three-layer polycaprolactone (PCL) -gelatin scaffold was designed by using different manufacturing methods (casting, electrospinning, freeze-drying), which effectively promoted wound healing and generated similar tissue to natural skin tissue in the SD rat model [41]. Similarly, porous prululam and gelatin composite scaffolds can recruit macrophages from the wound bed to the scaffold, leading to premature maturation of granulation tissue and a decrease in myofibroblasts in the Male C57BL/6 mouse model [42].

**Figure 1:**
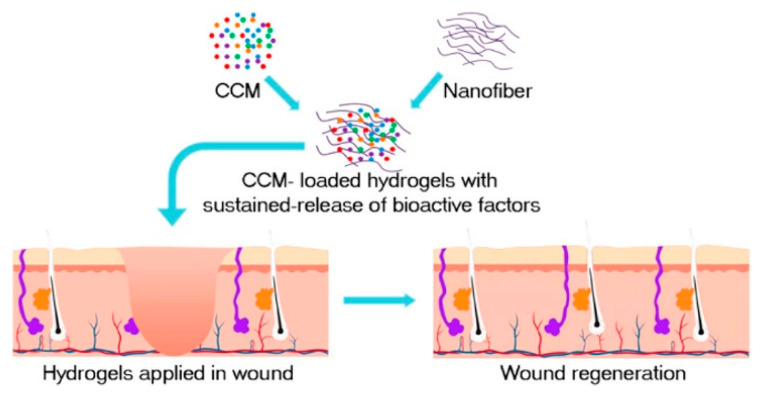
The preparation process of CMM-silk nanofiber composite hydrogel. The injectable hydrogel is used as a vehicle to load bioactive molecules secreted by the CMM, such as transforming growth factor-β1 (TGF-β1), insulin-like growth factor binding protein-1 (IGFBP-1), and platelet-derived growth factor-AB (PDGF-AB). Reprinted with permission from [46]. Copyright (2019) American Chemical Society.

SF occupies the dominant position in biomedical applications due to its “unique hierarchical structure, strong mechanical strength, good biocompatibility, and adjustable biodegradability, as well as abundant and low-cost sources” [43,44]. In the New Zealand rabbits ear model, the distribution of collagen fibers was more orderly and like normal skin after SF hydrogel treatment. The mean scar growth index was reduced by 16.6, with downregulation of α-SMA expression levels, suggesting that SF is an effective therapeutic agent to inhibit scar formation [45]. In C57 mice, an injected concentrated conditioned medium (CMM)–silk nanofiber composite hydrogel sustained released multiple bioactive factors and induced the proliferation and migration of fibroblasts, as shown in Figure 1 [46]. CMM was secreted by bone marrow mesenchymal stem cells (BMSCs).

**Figure 2:**
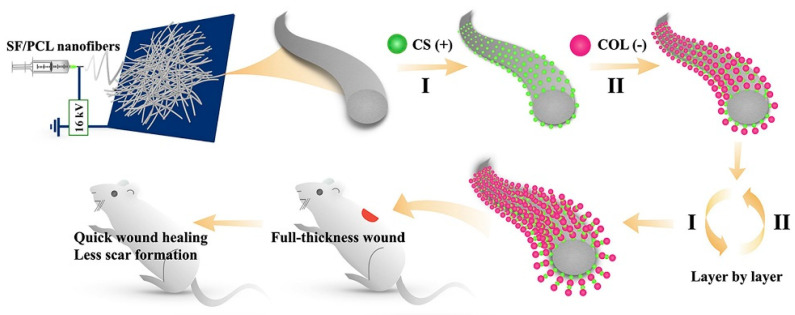
The preparation process of modified nanofibrous mats. The electrostatic spinning technique was applied to fabricate SF/PCL nanofiber mats. Positively charged CS and negatively charged collagen type I (COL) were deposited on the nanofiber mats by the layer-by-layer self-assembly technique. Reprinted with permission from [49]. Copyright (2020) Elsevier.

The SF/chitin/nanosilver composite scaffold exhibited excellent biocompatibility, blood coagulation, and antibacterial ability [47]. The silk three-dimensional scaffold with a “human skin structure similar” obtained through the carding-needling process, allowed human keratinous C4-I cells to grow normally on SF microfibers and guide the novo assembly of connective tissue, accompanied by thin collagen fibrils in the ECM through the C57BL/6 mouse model [48]. Nanofibrous mats containing SF and PCL, as well as CS and type I collagen, were prepared by a layer-by-layer self-assembly technique, as shown in Figure 2. In the Wistar rat model, the nanofibrous mats reduced wound closure time and scar formation through TGF-β/Smad signaling pathways [49]. Most tissue-engineered skin grafts have difficulty mimicking the complexity of natural ECM, porous ECM sponges were prepared by combining placental ECM with SF [50]. In the albino Wistar rat model, sebaceous glands, hair follicles, and thinner epithelium are highlighted, as placental ECM largely mimicked the internal environment of the wound and retained various cytokines/growth factors.

In summary, to promote wound healing and inhibit scarring, polysaccharide and protein materials from accurately mimic the components and structure of native ECM and induce cell adhesion, growth and differentiation. The addition of HA maintains the wetness of the lesion site, hopefully restoring the original microenvironment of the pre-traumatic tissue. CS is conducive to reduce wound inflammation due to its prominent and powerful antibacterial properties. Collagen scaffolds induce the orderly arrangement and normal secretion of collagen fiber and promote epithelialization and wound contraction. SF scaffolds are used to promote angiogenesis and reduce scar, accompanied by unique hierarchical structure, strong mechanical strength, and adjustable biodegradability. Notably, there are inevitable limitations for pure scaffolds on wound healing and scar inhibition, especially in hypoxic conditions where they do not function optimally.

## 3. Oxygen-Generating Scaffold

**Figure 3:**
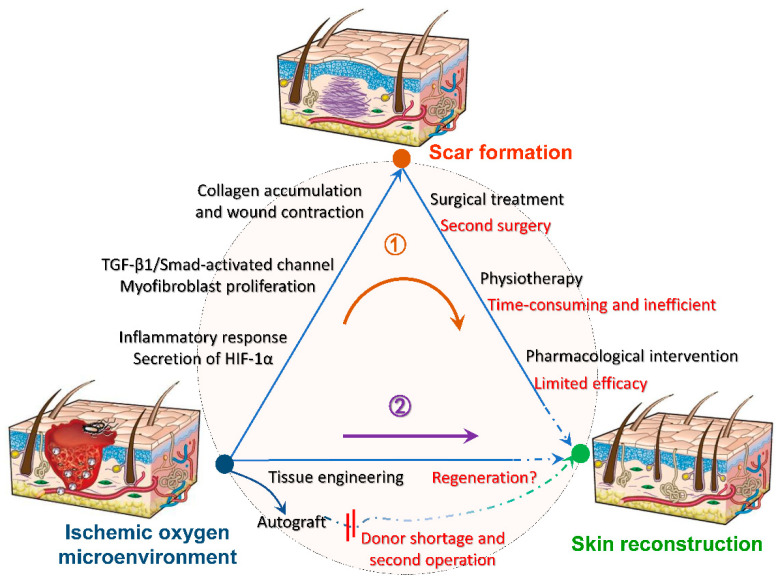
Mechanisms of oxygen-generating scaffolds to participate in trauma reconstruction. After hypoxia and anoxia of the damaged tissue, a series of reactions will contribute to scar formation, such as intense inflammatory response, conversion of fibroblasts to myofibers, excessive collagen deposition and wound contraction. Conventional treatments such as surgical excision are accompanied by limitations and incompleteness, making it difficult to heal the scar completely (route 1). Hence, we expect to intervene wound microenvironment by tissue engineering before scar formation to reduce or inhibit scar formation at the root (route 2). Reprinted with permission from [55]. Copyright (2008) Springer Nature.

Blood flow is the most important factor regulating the timeline of wound healing. Sufficient oxygen leads to the formation of new blood vessels, whereas hypoxia leads to cell apoptosis and exacerbated inflammatory response [51]. The high energy demand of inflammation and tissue reconstruction especially vascularization, accelerates wound hypoxia, creating a vicious circle [52]. Oxygen promotes the maturation of connective tissue, especially early tissue generation [53], while the secretion of hypoxia-inducible factor in hypoxia, prolongs the proliferation of excessive fibroblasts and the synthesis of collagen, as well as aggravates scar formations [54]. Furthermore, for traumatic injuries, adequate oxygen is a guarantee for the function of implanted scaffolds and is essential for cell survival. Further figure out the relationship between oxygen with tissue wound healing, various biomaterials are used as tissue scaffolds, among which oxygenated biomaterials are rare and used for skin are even rarer. The exploitation of biocompatible scaffolds that can continuously generate oxygen is expected to inhibit scar formation at the root, and its further mechanism is shown in Figure 3 [55]. As follows, a series of summaries of oxygen-generating biological scaffolds in different fields can be used as a reference to promote wound healing and inhibit scarring.

The ideal state involved in a dynamic oxygen generation system is [53,54]: an adequate supply of oxygen generation until new blood vessels are formed; appropriate rate of oxygen production, as too fast results in wasted oxygen and too slow is detrimental to maintaining cellular function; oxygen-producing materials and reaction residues are not harmful to the body. In general, oxygen-generating materials are based on high molecular weight polymer loaded with oxygen carriers or oxygen-producing compounds, to continuously supply oxygen to the lesion area. At present, in terms of oxygen sources, widely reported oxygen-generating materials are classified into three major categories: inorganic peroxides, perfluorocarbons (PFCs), and hydrogen peroxide (H_2_O_2_).

### 3.1. Inorganic Peroxide-Based Oxygen Generating Scaffold

Widely used inorganic peroxide materials include sodium percarbonate (Na_2_CO_3_), calcium peroxide (CaO_2_), and magnesium peroxide (MgO_2_), and among which CaO_2_ is the most used. Previous research has found that introduced Na_2_CO_3_ into polylactic acid-glycolic acid (PLGA) copolymer resulted in an observed release of oxygen within 24 h [56]. When the material was exposed to ischemic tissue, the rate of tissue necrosis and cell apoptosis decreased. It was speculated that oxygen release can delay tissue necrosis. The incorporation of CaO_2_ into PCL nanofibers produced H_2_O_2_ and calcium hydroxide when exposed to water, which inhibited the growth of Gram-positive and Gram-negative bacteria [57]. Although there was certain cytotoxicity to human osteoblasts during the outbreak period when the concentration of H_2_O_2_ was increased, cells could recover after 4 days, suggesting that the material could avoid long-term toxicity. Gelatin methacryloyl hydrogels loaded with CaO_2_ released oxygen for more than 5 days, reducing hypoxia-induced necrosis [58]. A new core–shell structure oxygen-generating scaffold was synthesized from human keratin, SF, gelatin, and CaO_2_, in which the CaO_2_ was the core embedded in the scaffold and gelatin was the shell with stably high levels of oxygen release within 14 days in vitro [59]. When the 3D-printed scaffolds coated with CaO_2_ were encapsulated within the PCL matrix, oxygen release was sustained and dependent on the concentration of CaO_2_ [60]. Sustainable oxygen release also occurred at antioxidant polyurethane-CaO_2_ cryogel scaffolds prepared by freeze-drying technique, which promoted the survival of H_9_C_2_ cells during hypoxic conditions [61].

These findings provided solid evidence that inorganic peroxide oxygen-generating scaffolds have achieved remarkable achievements in generating oxygen and supporting cell survival. However, these scaffolds cannot change their own limitations and avoid the damage of the reaction leftovers to the internal host tissues. In brief, inorganic peroxides may lead to cause an imbalance in metal levels in the body, making it difficult to circulate oxygen in the blood which may cause irreversible damage to cells [51].

### 3.2. PFCs-Based Oxygen Generating Scaffold

PFCs, which are approximately 20 times more soluble in oxygen than water, are typically combined with scaffold materials to create a homogeneous oxygen environment in three-dimensional tissue structures. Among them, the introduction of 7% perfluorooctyl bromide showed a threefold increase in oxygen permeability [62]. A scaffold system with hollow particles containing fluorooctane emulsion, produced oxygen in time for cells attached to the hollow particles, to prevent cell necrosis in a hypoxic environment until neointima formation [63]. Embedding fluorinated zeolite particles in a three-dimensional polyurethane scaffold significantly increased cell viability than the control, and dissolved oxygen concentration increased with increasing fluorinated zeolite [64]. However, fracture stress and mechanical strength decreased dramatically with the addition of PFCs in the scaffold material, suggesting that this was not sufficient to provide adequate support for cell adhesion and migration [65].

### 3.3. H_2_O_2_-Based Oxygen Generating Scaffold

**Figure 4:**
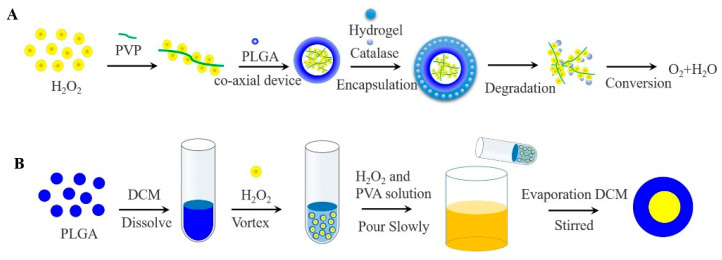
(**A**) Oxygen generating systems were fabricated via the coaxial device, with PLGA as the outer shell, H_2_O_2_ and PVP complexes as the core. While the released H_2_O_2_ was converted to oxygen and water by contacting with catalase in the thermosensitive hydrogel. (**B**) Schematic of the W_1_/O/W_2_ method for the preparation of PLGA-H_2_O_2_ microspheres.

In contrast to inorganic peroxide and PFCs oxygen-generating materials, H_2_O_2_ produces only molecular oxygen and water under the catalysis of the catalase (CAT), avoiding the toxicity of the reaction leftovers and the imbalance of body ions. CAT has abundant sources, good biocompatibility, and low immunogenicity. In general, H_2_O_2_ has usually encapsulated in a bilayer even more layers of high molecular weight polymer to avoid direct contact with cells and maintain cellular viability [66]. The rate of oxygen generates is also regulated by the degradation of high molecular weight polymer. Li constructed an oxygen release system consisting of PLGA-H_2_O_2_ microspheres and thermosensitive hydrogel, which allowed the release of oxygen for more than 14 days [67]. In brief, it was prepared by a coaxial device, with PLGA as the outer shell, H_2_O_2_ and poly (2-Pyridone) (PVP) complexes as the core. As the outer thermosensitive hydrogel system degrades, the CAT and H_2_O_2_ met each other and generated oxygen and water, avoiding direct contact with cells to cause toxicity, as shown in Figure 4A.

Others, H_2_O_2_ encapsulated in PLGA microspheres by applying double-emulsification solvent evaporation method (W_1_/O/W_2_), oxygen can be monitored within 5 h [68]. Concretely, PLGA was dissolved in methylene chloride to form the W1 phase, H_2_O_2_ was added to form the O phase, and the first W_1_/O emulsion was formed after high-speed stirring. Then the emulsion was poured into an aqueous phase (W_2_) containing polyvinyl alcohol and H_2_O_2_ to obtain a W_1_/O/W_2_ emulsion. Finally, the final emulsion was continuously stirred to evaporate the methylene chloride, and filtered to collect PLGA microspheres containing H_2_O_2_, as shown in Figure 4B. A layer of alginate pre-cured with CAT was applied to the surface of PLGA microspheres to form a bilayer system, with the result that oxygen continued to be released when H_2_O_2_ was exposed. The cell survival was significantly improved especially when 4% H_2_O_2_ was contained [69].

Rajendar prepared microcapsules of polymethyl methacrylate loaded with H_2_O_2_-H_2_O [70]. The inner part of microcapsules, “H_2_O_2_-H_2_O_2_” droplets are continuously and randomly distributed. The encapsulated H_2_O_2_ diffused out of the microcapsule at a fixed rate and decomposed into water and molecular oxygen, with 70% of the H_2_O_2_ releasing oxygen within 24 h. Polymethyl methacrylate, a biomedical polymer approved by FDA, has good biocompatibility. Interestingly, by 2020, PLGA microspheres encapsulating sorafenib and CAT were prepared by the W_1_/O/W_2_ method. CAT generated oxygen by reacting with H_2_O_2_ in vivo [71].

In summary, a continuous and controlled oxygen supply to damaged tissues facilitate cell survival and scaffold functionalization before angiogenesis and accelerates tissue reconstruction from the root. In the field of tissue engineering, the research of oxygen-generating scaffolds has made significant progress, which will provide theoretical support and great promise for scarless healing.

## 4. Scaffolds with Stem Cells or Growth Factors

### 4.1. Stem Cell-Seeded Scaffolds

Wound healing is a complex process involving the interaction of relevant matrixes, cells, factors, etc. In tissue engineering, mesenchymal stromal cells (MSCs), accompanied by super self-renewal and differentiation abilities, are participated in promoting wound healing and inhibiting scar formation after targeted delivery to the site of traumatic defects using scaffolds. MSCs possess immunomodulatory properties and regulate the expression and secretion of growth factors such as VEGF, epidermal growth factor (EGF) [72]. Three prominent MSCs in wound tissue reconstruction are dominated by BMSCs, adipose stem cells (ADSCs), and placenta-derived mesenchymal stem cells (PMSCs). Notably, exosomes, the main product of the paracrine action of MSCs, in recent years, have highlighted a positive effect on wound healing and scar inhibition and serve as theoretical support for the role of MSCs [73].

BMSCs are derived from bone marrow stems, with the ability to transform into effector cells such as keratinocytes or endothelial cells, widely as a potential candidate cell for scarless healing [74,75]. BMSCs regulate the wound microenvironment by sensing the levels of inflammatory factors, releasing growth factors such as VEGF, EGF [76]. In addition, BMSCs also down-regulate TGF-β1, type I collagen, and α-SMA to remodel the ECM [77]. The conditioned medium of BMSCs contributes to attenuate fibrosis [78]. In the C57 mouse model, thermosensitive hydrogel loaded with BMSCs exhibited faster tissue remodeling by promoting the value-added of keratin-forming cells, TGF-β1 secretion, etc. [79]. Injectable silk hydrogels encapsulated with BMSCs resulted in faster defect healing and many hair follicles than controls in SD rats [80]. Inoculation of BMSCs onto collagen-tussah SF hybrid scaffolds prepared by freeze-drying method showed moderate deposition and orderly arrangement of collagen fibers in the SD rat model, which is beneficial for preventing scar tissue formation [81]. Aloe/BMSCs hybrid scaffold and CS/BMSCs hybrid scaffold accelerated wound healing because of enhanced vascularization after implantation in Wistar-albino male rats [82]. However, other researchers reported that it may aggravate scar formation when it refers to heterogeneity, dose, application route, timing, and donor source of BMSCs [83].

ADSCs, which have similar properties to BMSCs, are readily available from adipose tissue and do not involve ethical issues. [84]. ADSCs could restrain inflammation by promoting the release of M2-type macrophages (anti-inflammatory), accelerate angiogenesis and remodel the cytoplasmic matrix by secreting VEGF [85]. Direct intradermal injection of ADSC into a rabbit ear hyperplastic scar model, regular collagen fiber arrangement and less scar formation was observed, which was speculated to be formed due to a decrease in α-SMA and type 1 collagen expression [86]. In the BALB/c male mouse model, typical hair follicle structures were observed when ADSCs were inoculated onto decellularized amniotic scaffolds [87]. Decellularized human amniotic membrane/electrospun nanofiber scaffolds seeded with ADSCs were applied to the BALB/c male mouse model. By reducing inflammation and inducing matrix metalloproteinases secretion, the scaffold promoted collagen degradation during remodeling and reduced scar formation with a scar height index of 1.1 ± 0.12. The smaller the index, the smaller the scar formation and the scar height index for normal skin was 1 [88].

Compared with BMSCs and ADSCs, PMSCs survive better in a hypoxic environment. PMSCs culture medium in hypoxic conditions can inhibit the excessive proliferation and migration of fibroblasts in vitro experiments [89]. In the female NMRI-Foxn1nu/Foxn1nu mouse model, three umbilical cord-derived PMSCs (Amnion-derived MSCs, blood vessel-derived MSCs from the chorionic plate, and Wharton’s jelly-derived MSC), all promoted vascularization and wound healing. Among them, the dermal substitute Matriderm^®^ served as a vehicle for rapid delivery of PMSCs to the defect site [90].

Exosomes with a diameter of 30–100 nm, which are secreted from stem cells, exhibit a lower immunogenic response and are easier to maintain bioactivity during storage than stem cells [91]. Exosomes derived from human umbilical cord mesenchymal stem cells were encapsulated in polyvinyl alcohol/alginate nano hydrogel, with the results showed that it contributed to angiogenesis and wound healing in SD rats by activating the ERK1/2 pathway [92]. In the Wistar rat model, infiltration of exosomes from ADSCs with sodium alginate hydrogel enhanced wound closure and angiogenesis, with a wound closure percentage of 94.80 ± 1.07% at 14 days [93]. Similarly, high porous cryogels composed of antioxidant polyurethane, after being supplemented with exosomes derived from ADSCs, showed complete wound closure and complete mature epithelial structures with hair follicles and glands in Wistar rats [94].

To sum up, scaffolds as vehicles for the delivery of BMSCs, ADSCs, and PMSCs to specific sites are all effectively involved in the reconstitution of the ECM and demonstrated the potential to promote wound healing and inhibit scar formation. Exosomes as a core target of stem cell action, further elucidate the mechanisms of positive stem cell effects on trauma and suggest another hot trend in tissue regeneration.

### 4.2. Growth Factors-Loaded Scaffolds

The reconstruction of damaged tissues is dependent heavily on the coordinated interplay in space and time of different growth factors which can modulate cell behavior [95]. The half-life of growth factors in vivo is only a few minutes, with poor stability and rapid degradation, while repeated supply may cause adverse reactions [96]. Therefore, to obtain a stable release rate, it makes sense to deliver growth factors to specific wound sites via scaffolds. Some significant growth factors will be mentioned, such as bFGF, VEGF, EGF, and PDGF-BB.

Hydrogel dressings encapsulated with heparin and bFGF showed less inflammation and higher expression of VEGF in the early stages of wound healing. The encapsulated heparin was used to stabilize bFGF and control the rate of bFGF release [97]. Electrospun core-sheath fibers loaded with bFGF in the male SD rat model showed higher wound healing rates and vascularization, and that the collagen fiber arrangement and composition were similar with normal tissue [98]. In addition, the therapeutic effects of bFGF on SD rat wound defect model, rabbit ear model of hypertrophic scarring and human scar fibroblast models were investigated, showing the promise of bFGF to reduce scarring by increasing the secretion of matrix metalloproteinases-1 [99]. In addition, bFGF was demonstrated to reduce scar formation by inhibiting the differentiation of epidermal stem cells to myofibroblasts [100]. VEGF acts as critical molecular targets to increase vascular permeability and promote vascularization [101]. After VEGF and BMSCs were co-embedded in a 3D gel prepared from fibrinogen and thrombin, the defective area exhibited intact dermis and epidermis after 3 days in the SD rat model [102]. PDGF acts primarily as a chemo-attractant for the neutrophils and fibroblasts [103]. VEGF, PDGF-BB, and TGF-β1 were combined with alginate-sulfate, resulting in that the percentage of blood vessel density and vascular maturity was threefold greater than the untreated scaffold in the SD rat model [104]. Notably, scaffolds loaded with VEGF provide clear benefits, but the effects of dose, time, and space distribution of VEGF on angiogenesis remain complex and even unknown [105]. What is more, the scarless phenotype can be converted to a scar-forming phenotype by adding exogenous VEGF [106]. Polysaccharide hydrogels based on CS /alginate were used to deliver EGF, and the results confirmed that the porous gels were conducive to the loading and release of EGF, and promoted cell proliferation and wound healing in the SD rat model [107].

As mentioned above, different scaffolds were used as mediators to transport growth factors such as bFGF, VEGF, EGF, and PDGF-BB to the site of action, which was used to prolong the survival time of growth factors and intervene wound healing effect and scar formation.

## 5. Summary and Prospects

Scars are initiated by tissue defects as a result of ischemia and hypoxia, which in turn produce a series of issue self-healing behaviors such as severe inflammatory reactions. Although promising advances have been made in wound repair and scar treatment fields, the mechanisms of scar development and inhibition remain unclear. Conventional scar repair methods are compensatory treatments after scar formation, making it difficult to remove scars and restore the structure and function of the normal skin. What is worse, most of these may cause secondary damage to the wound or cause serious complications. Therefore, useful measures should be taken to intervene in the early stages of the wound to reconstruct a favorable microenvironment in the lesioned area and to guide the arrangement and deposition of the collagen matrix. Normal wound healing and reduced scar formation are always characterized by a minimal inflammatory response, a large amount of HA and neovascularization.

Based on bionic principles, tissue-engineered artificial skin should contain sufficient polysaccharides and proteins, oxygen, and nutrients in the early stages of grafting, which will contribute to reduce the exacerbation of inflammation caused by hypoxia and ischemia, reduce the overexpression and disorganization of rough and hard collagen bundles. In this review, various scaffold materials are discussed, from polysaccharide scaffolds to growth factor scaffolds. Overall, polysaccharide scaffold and protein scaffold highly mimic the function of skin tissue in terms of shape, structure, function, and mechanical strength. Stem cell-seeded scaffolds or growth factors-loaded scaffolds, with the secretion of bioactive substances, highlight the potential to promote wound healing and inhibit scarring. Furthermore, the exosomes scaffold (the main component of stem cells) further explains the mechanism of action of stem cells in reverse. Adequate oxygen facilitates the promotion of tissue angiogenesis and wound healing, which is fundamental for cell survival and crucial for the success of scaffold grafts. In this sense, as mentioned previously, in the field of tissue engineering, the staged success of research on oxygen-generating scaffolds will offer great prospects for scarless wound healing.

Therefore, we believe that future tissue-engineered scaffolds should contain protein and polysaccharide components and be able to provide nutrients and seed cells while continuously releasing the ideal amount of oxygen, allowing the scaffold to accelerate wound vascularization and reduce excessive collagen deposition.
